# An outbreak of aseptic meningitis caused by coxsackievirus A9 in Gansu, the People's Republic of China

**DOI:** 10.1186/1743-422X-7-72

**Published:** 2010-04-06

**Authors:** Aili Cui, Deshan Yu, Zhen Zhu, Lei Meng, Hui Li, Jianfeng Liu, Guiyan Liu, Naiying Mao, Wenbo Xu

**Affiliations:** 1 National Institute for Viral Disease Control and Prevention, Chinese Center for Disease Control and Prevention, 27 Nanwei Road, Beijing 100050, PR China; 2 Gansu Center for Disease Control and Prevention, 12 Donggangxi Road, Lanzhou 730030, PR China; 3 Jilin Center for Disease Control and Prevention, 35 Gongnongda Road, Changchun 130021, PR China

## Abstract

**Background:**

An outbreak of aseptic meningitis occurred in Tianshui city of Gansu Province, the People's Republic of China, from March to June 2005. A total of 85 patients were clinical confirmed as aseptic meningitis in this outbreak.

**Results:**

CVA9 was mainly responsible for this outbreak supported by the clinical manifestations of the patients, epidemiological data of the outbreak, the results of RT-PCR and complete VP1 sequence determination, conventional neutralization assays, IgM serological assays, viral isolation and phylogenetics analysis. Through phylogenetic analysis and homogeneity analysis for partial VP1 gene, the nucleotide and amino acid homologies between Gansu isolates and former Chinese CVA9 strains were 88.2%-96.1% and 97.2%-99.2%, respectively. Multiple transmission chains of CVA9 occurred in different provinces or years in China. Moreover, in order to clarify the genotype of CVA9, Gansu CVA9 strains isolated in this outbreak were compared with other CVA9 isolates based on VP1/2A junction regions (genotyping region) and they might belong to a new genotype of CVA9, which could be assigned for genotype XIII,

**Conclusions:**

CVA9 was confirmed as the pathogen responsible for this outbreak. The phylogenetic analysis indicated that the CVA9 strains isolated in this outbreak might belong to a new genotype.

## Background

Human Enteroviruses (HEV) comprise more than 80 distinct serotypes within the Picornaviridae family, and are currently classified into five species: Poliovirus and HEV-A, HEV-B, HEV-C, and HEV-D [1]. Most infections are asymptomatic, but enteroviruses can cause a wide variety of clinical diseases, the most common of the potentially severe enterovirus disease in pediatric patients remains aseptic meningitis. Serotypes of the HEV-B species including CVB1-6, ECHO, CVA9, HEV69 and HEV73 are the most frequently implicated [2-6], which could cause sporadic cases, outbreaks or epidemics of aseptic meningitis worldwide including China [6].

In China, the repeated outbreaks of HEV-associated diseases occurred in recent years, and these patients were reported to have an aseptic meningitis symptom were usually caused by HEV-B [7,8]. Here, we describe an outbreak of aseptic meningitis in Tianshui city of Gansu province, China, from March to June 2005. The etiological agent responsible for this outbreak was identified and the genetic characterizations of virus strains that were isolated from this outbreak were also analyzed.

## Methods

### The collections of clinical specimens in this outbreak

In this outbreak, the clinical specimens were collected from 22 out of 85 patients during Apirl to June. Like other 63 cases, these 22 patients came from Maiji district of Tianshui city were hospitalized cases and confirmed as aseptic meningitis when these patients were admitted to the hospital. So it clearly showed the epidemiological linkage between 22 patients and 63 other patients.

10 cerebrospinal fluids (CSF), 22 stool samples and 22 serum samples were collected from 22 patients with clinical confirmed aseptic meningitis during acute-phase infection after onset; 3 patients were also found to have convalescent sera. A total of 32 etiology specimens and 25 sera obtained during this outbreak were stored at -80°C and -20°C, respectively, for further analysis.

### Viral RNA extraction, direct reverse transcription-polymerase chain reaction and sequences determination

Viral RNA was directly extracted from the clinical specimens using a QIAamp Mini Vial RNA Extraction Kit (Qiagen, Valencia, CA, USA). 10 CSF specimens and 22 stool samples from 22 patients was performed with direct reverse transcription-polymerase chain reaction (RT-PCR) by the pan-enterovirus primer pair including primer PE1 and PE2 as previously described [9].

### Virus Isolation and identification

10 CSF specimens and 22 stool samples were separately inoculated into human rhabdomyosarcoma (RD) cell lines and were cultured in a maintenance medium at 36°C. Cultures that exhibited a characteristic enterovius cytopathic effect (CPE) were identified by RT-PCR and sequencing with the following method. If no CPE was observed in the cultures, these were further cultured for another 7 days. Viral RNA was extracted from culture with CPE by using the QIAamp mini viral RNA extraction kit (Qiagen, Valencia, CA, USA). The entire VP1 gene was amplified by RT-PCR with the primer pairs including the previous reported primer pair 008 and 011 [2]. The amplification products were purified using a QIAquick Gel Extraction Kit (Qiagen), and the amplicons were bidirectionally sequenced using an ABI PRISM 3100 Genetic Analyzer (Applied Biosystems, Hitachi, Japan).

### Identification of the genotype of CVA9

Though entire VP1 region were obtained for serotyping of human enterovirus, few sequences were available at GenBank, so it is difficult to analyze the genetical linkage of sequences between China and other countries. In order to clarify the genotype of CVA9, the genotyping region (VP1/2A region) was also amplified with the previous reported primer pair named primer VP+ and 2A- [10]. The RT-PCR products were purified and sequenced with the above mentioned method.

### Sequence Analysis

The sequence data were aligned, edited, and assembled to obtain the entire VP1 gene and VP1/2A region using Sequencher version 4.0.5 (GeneCode, Ann Arbor, MI, USA). Sequence alignments were created with ClustalX software 1.81, the pairwise sequence identities of the nucleotide and deduced amino acid sequences were calculated with BioEdit software 7.0. The genetic distances were analyzed with Kimura 2-parameter method of MEGA 4.0 program, and neighbor-joining of phylogenetic analysis were used to provide a more reliable inference of phylogeny with MEGA 4.0 program.

### Serology detection of Coxsackievirus IgM antibody

22 acute-phase sera collected from the clinical confirmed cases within 2-29 days after onset were detected Coxsackievirus immunoglobulin M (IgM) antibodies using an Serion ELISA classic Coxsackievirus IgM test kits (Institute Virion/Serion GmbH, Wurzburg, Germany). This kit is quantitative and qualitative tests for detection of human antibodies in serum or plasma against all serotypes of Coxsackievirus.

### Neutralization Test

In this study, according to the methods previously described [11], conventional neutralization test was also performed with three pairs of paired serum samples, and the entire virion of strain Gansu05-1/GS/CHN/2005 isolated in this outbreak and identified was used as the neutralization virus and the 50% cell infectious dose (CCID_50_/50 μl) was calculated according to the Kärber formula [12].

### Nucleotide sequence accession numbers

The nucleotide sequence of the complete VP1 gene and VP1/2A region for representative strain Gansu05-1/GS/CHN/2005, which was determined in this study, has been deposited in the GenBank nucleotide sequence database under accession number GQ294574 and GQ294575, respectively.

GenBank accession numbers for each human enterovirus were shown as follows: CVA9(D00627), CVA12(AF081302), CVA16(U05876), CVA19(AF081308), CVA24(AF081311), CVB1(M16560), CVB2(AF081312), CVB3(M16572), CVB4(D00149), CVB5(X67706), CVB6(AF081313), E1(AF081314), E2(AF081315), E3(AF081316), E4(AF081317), E5(AF081320), E6 (AF081321), E7(AF081324), E9(X92886), E11(X80059), E12(X79047), E13(AF081327), E14(AF081328), E15(AF081329), E16(X89545), E17(AF081330), E18(AF081331), E19(AF081332), E20(AF081333), E21(AF081334), E24(AF081335), E25(AF081336), E26(AF081337), E27(AF081338), E29(AF081339), E30(AF081340), E31(AF081344), E32(AF081345), E33(AF081346), EV68(AF081348). GenBank accession numbers for each CVA9 were shown as follows: Griggs(D00627), 06.109.3344(FJ868282), FJ00-127(AY573578), FJ98-90(AY573577), 78-97(AB268124), 49-98(AB268123), 148-00(AB268121), 03-171FCR2(AB167980), 03-144NPC3(AB167979), 03-132NPR2(AB167978), YZ047/SD/CHN/2005/CA9(GQ246517), 04318/SD/CHN/2004/CA9(GQ329731), 01332/SD/CHN/2001/CA9(GQ329730), 00365/SD/CHN/2000/CA9(GQ329729), 97186/SD/CHN/1997/CA9(GQ329728), 97089/SD/CHN/1997/CA9(GQ329727), TR081056-05(AM236967), Can-1-85 (AF166250), Fin-2-95(AF166251), Net-1-79(AF166252), Net-1-64(AF166253), Net-1-81(AF166249), Fin-1-88(AF166248), Den-1-71(AF166247), Net-1-59(AF166246), Net-1-63(AF166245), Net-1-67(AF166244), Net-1-61(AF166243), Mex-1-75(AF166242), Hon-1-77(AF166241), US-CO-1-74(AF166240), US-MD-1-88(AF166239), Can-1-86(AF166238), US-NC-1-83(AF166237), Net-1-71(AF166236), Net-1-78(AF166235), Thai-1-93(AF166234), Net-1-73(AF166233), US-MS-1-78(AF166232), Net-2-79(AF166231), Fin-2-83(AF166230), Fin-1-83(AF166229), Fin-2-97(AF166228), Fin-1-97(AF166227), Fin-1-96(AF166226), Fin-2-96(AF166225), Fin-3-96(AF166224), Fin-2-94(AF166223), Fin-1-94(AF166222), Fin-1-95(AF166221), Fin-2-93(AF166220), Fin-1-93(AF166219).

## Results

### Epidemiological investigation of this outbreak

**Figure 1:**
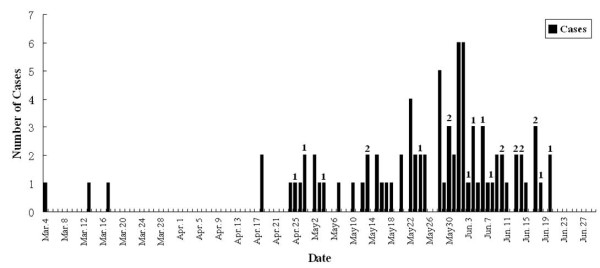
**Distribution of aseptic meningitis cases in Tianshui city of Gansu Province by date**. On March 4, 2005, the first case appeared. The number of similar cases had increased dramatically. By June 29, 2005, the outbreak had affected 85 children. The numbers above column indicated 22 out of 85 patients in this outbreak.

On 4 March 2005, a child was clinical diagnosis as aseptic meningitis in Tianshui city of Gansu Province. Subsequently, the number of cases with clinical confirmed aseptic meningitis increased rapidly. Up to 29 June 2005, a total of 85 cases, which concentrated in Maiji district of Tianshui city of Gansu province, were clinical confirmed as aseptic meningitis by Tianshui City People's Hospital in this outbreak (Figure 1). Also through the epidemiological investigation, no similar case was reported in other districts of Tianshui city.

The clinical symptoms of the patients were fever (>37.5°C) (79/79, 6 cases missing), headache (67/85) and vomiting (56/85). The incidence of the relevant cases peaked in late May to early June. All patients were less than 13 years old, and a majority of these patients were 4-5 years old. Numbers of Gregarious children's cases (from kindergarten and primary school) were more than those of scattered children. All patients had good prognosis, and no fatal case was found in this outbreak.

### Laboratory confirmation of CVA9 as the pathogenic agent in this outbreak

The specimens collected from 22 patients were identified by pan-entero RT-PCR, virus isolation/molecular typing, IgM ELISA and NT, and the results were showed in the table 1.

**Table 1:** Laboratory confirmation of the pathogenic agent in this outbreak*

Cases	Molecular detection				Serology detection		
	
	Direct RT-PCR for pan-enterovirus		Virus isolation/serotyping		ELISA testing for Coxsackievirus IgM of 22 acute-phase sera	Neutralization Test	
			
	CSF	Stool	CSF	Stool		Neutralization titer in acute-phase	Neutralization titer in convalescent
1	+	+	+/CVA9	+/CVA9	+	<1:20	1:501
2	+	+	-	-	+	<1:20	1:1000
3	+	+	-	+/CVA9	+	<1:20	>1:1024
4	+	/	+/CVA9	/	-	/	/
5	-	+	-	+/CVA9	+	/	/
6	-	+	-	+/CVA9	+	/	/
7	-	+	-	+/CVA9	+	/	/
8	-	+	-	+/CVA9	+	/	/
9	-	+	-	-	+	/	/
10	-	+	-	-	-	/	/
11	/	+	/	-	-	/	/
12	/	+	/	-	-	/	/
13	/	+	/	-	-	/	/
14	/	-	/	-	+	/	/
15	/	-	/	-	+	/	/
**Total**	**4**	**12**	**2//CVA9**	**6//CVA9**	**10**	**At least four times higher for all three pairs of paired serum samples**	

* this table only showed the positive results of 22 cases in this outbreak;

/ showed no specimens obtained in this outbreak.

4 CSF sample and 12 stool samples (including 3 CSF samples and 3 stool samples detected from 3 patients) were successfully amplified with pan-enteroviruse primer pair. It indicated the enteroviruse infection responsible for this outbreak.

Among them, 8 samples including 2 CSF and 6 stool samples were positive for virus isolation/VP1 RT-PCR, and the other 8 samples were negative and only positive for pan-enteroviruse directly RT-PCR, this maybe due to the lower titer of the virus in the clinical samples. As for the viral isolation, 2 CSF and 6 stool samples out of 32 samples (including one CSF and one stool sample from one patient) were observed with the characteristic enterovirus CPE, which appeared within two passages after inoculation, and the entire VP1 was successfully amplified to obtain the predicted products of 906 bp.

**Figure 2:**
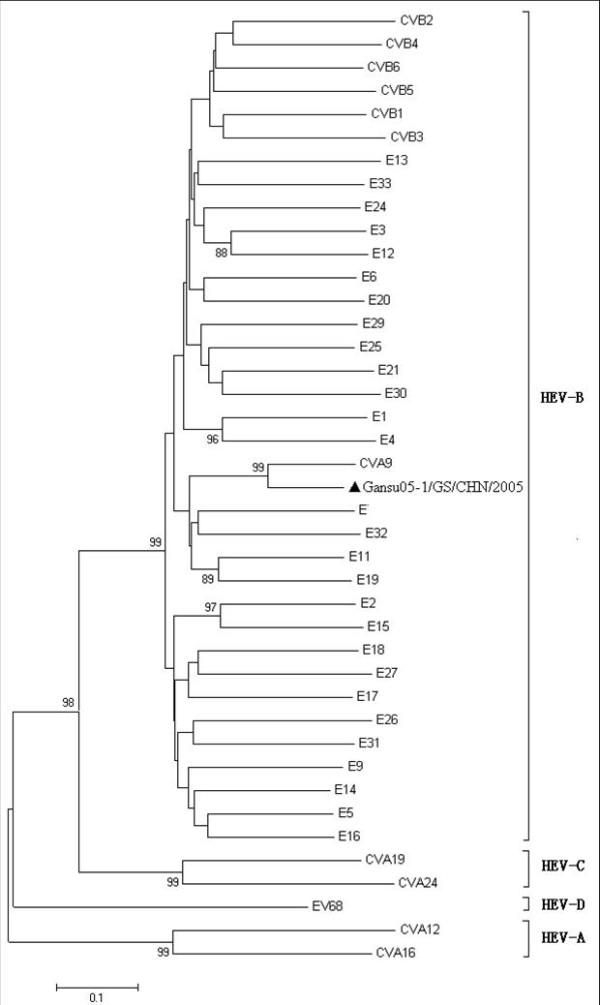
**Dendrogram illustrating sequence relationships among the studied CVA9 isolates and the prototype strains of Human enterovirus**. The phylogenetic tree constructed based on analysis of VP1 gene was drawn by bootstrap analysis (500 times) using the Neighbor-joining of MEGA 4.0 software. The triangle in the figure showed strain Gansu05-1/GS/CHN/2005 was sequenced in this study.

Sequence determination revealed that eight viral isolates exhibited 99.9% to 100% homology in their nucleic acid sequences. Therefore, the data showed that all the patients were infected with the similar virus. A viral isolate designated as strain Gansu05-1/GS/CHN/2005 was used for phylogenetic analysis. Furthermore, phylogenetic tree was conducted on the basis of the entire VP1 sequences of human enterovirus prototype strains indicated that the sequences was very close to the prototype strain of CVA9 (strain Griggs) (Figure 2).

In ELISA detection for Coxsackievirus IgM, 10 sera obtained positive results with the proportion of positive samples being 45.5%. This result also supported that Coxsackievirus infections occurred in this outbreak.

Conventional neutralization test results revealed that the neutralization titers of the convalescent-phase samples were at least four times higher than those of the acute-phase samples for all three pairs. This finding also suggested that CVA9 was associated with this outbreak.

### Phylogenetic analysis of CVA9

**Figure 3:**
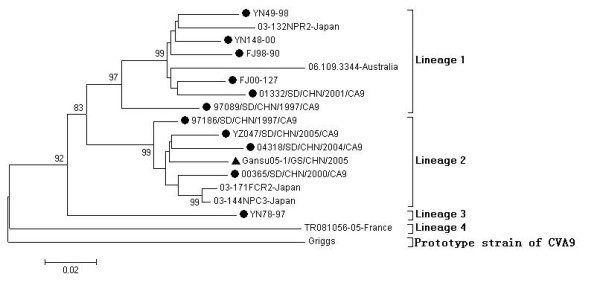
**Dendrogram illustrating sequence relationships among the studied CVA9 isolates and the prototype Griggs strain based on VP1 gene**. The triangle in the figure showed strain Gansu05-1/GS/CHN/2005 was sequenced in this study. The circle in the figure showed CVA9 strains isolated in China.

Only the partial VP1 gene (nt2583-nt3338, relative to strain Griggs) of former Chinese CVA9 strains was available in the GenBank database. Thus, in order to determine the molecular epidemiology of CVA9 in China, the phylogenetic dendrogram (Figure 3) was conducted with 12 Chinese CVA9 strains, 5 strains from other countries and the prototype strain based on this region. 12 Chinese CVA9 strains (including strain FJ98-90 and FJ00-127 from Fujian province, strain YN148-00, YN49-98 and YN78-97 from Yunnan province, strain 01332/SD/CHN/2001/CA9, 97089/SD/CHN/1997/CA9, 97186/SD/CHN/1997/CA9, YZ047/SD/CHN/2005/CA9, 04318/SD/CHN/2004/CA9 and 00365/SD/CHN/2000/CA9 from Shandong province) were isolated during 1997 to 2005; 5 strains from other countries composed of 3 Japanese strains (stain 03-171FCR2, 03-144NPC3 and 03-132NPR2) isolated in 2003, one strain (strain 06.109.3344) isolated in Australia in 2006 and one strain (strain TR081056-02) isolated in France in 2005. Except the prototype strain, 17 CVA9 strains could be divided into 4 different lineages (lineage 1-4). 10 strains including 8 Chinese strains (YN148-00, YN49-98, FJ98-90, FJ00-127, 01332/SD/CHN/2001/CA9 and 97089/SD/CHN/1997/CA9), one Japanese strain (strain 03-132NPR2) and one Australian strain (strain 06.109.3344) belong to lineage 1; 7 strains including 5 Chinese strain (Gansu05-1/GS/CHN/2005, 97186/SD/CHN/1997/CA9, YZ047/SD/CHN/2005/CA9, 04318/SD/CHN/2004/CA9 and 00365/SD/CHN/2000/CA9) and two Japanese strains (strain 03-171FCR2 and 03-144NPC3) belong to lineage 2; strain YN 78-97 from China form lineage 3; and strain TR081056-02 from France form lineage 4.

The genetic distances between and within lineages were 0.11-0.21 and 0.04-0.07, respectively. Chinese CVA9 strains were concentrated in lineage 1, 2, and 3. The result of the phylogenetic tree indicated that multiple transmission chains occurred in different provinces or years. The nucleotide and amino acid homologies between strain Gansu05-1/GS/CHN/2005 and other Chinese CVA9 strains within this partial VP1 gene were 88.2%-96.1% and 97.2%-99.2%, respectively. And strain Gansu05-1/GS/CHN/2005 isolated in this outbreak was the closest to strain 97186/SD/CHN/1997/CA9 isolated in Shandong province in 1997.

**Figure 4:**
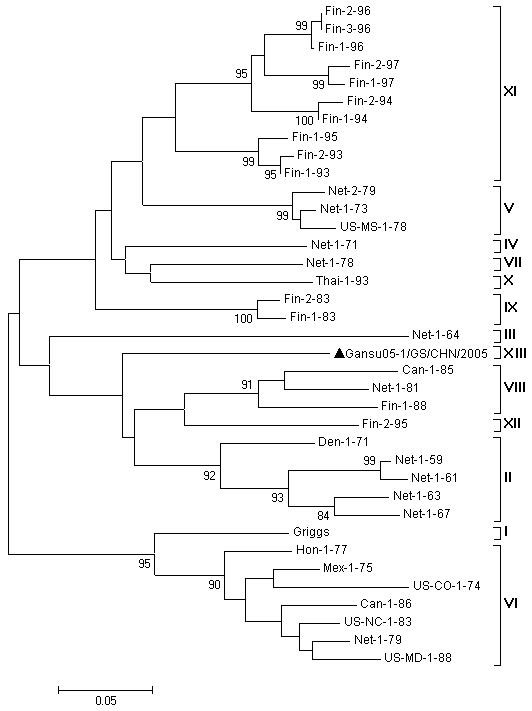
**Dendrogram illustrating sequence relationships among the studied CVA9 isolates and the prototype Griggs strain based on VP1/2A junction region**. The triangle in the figure showed strain Gansu05-1/GS/CHN/2005 was sequenced in this study.

Due to few VP1 sequences were available at GenBank, in order to investigate the genetic relationships between strain Gansu05-1/GS/CHN/2005 and other CVA9, phylogenetic analysis were conducted on the basis of the sequences in the junction region between the VP1 capsid protein and the 2A protease genes (VP1/2A, nt3258-nt3407, relative to strain Griggs) with 37 CVA9 strains (Figure 4). CVA9 genotypes have been reported to define as clusters of related strains with <15% nucleotide divergence in this region [10], and CVA9 strains could be assigned to 12 genotypes, which were designated genotype I-XII in the chronological order of the isolation of the earliest virus strain in each genotype [10]. In this study, the 24.0% nucleotide divergence was found between strain Gansu05-1/GS/CHN/2005 and the prototype strain Griggs; compared with the genotype I-XII of CVA9 strains, the nucleotide divergence of strain Gansu05-1/GS/CHN/2005 were 17.3%-32.6%. Moreover, strain Gansu05-1/GS/CHN/2005 formed an independent branch in the phylogenetic tree compared with other genotypes of CVA9 (Figure 4). So, both the results of phylogenetic analysis and nucleotide divergence indicated that strain Gansu05-1/GS/CHN/2005 might be a new genotype of CVA9, and could be assigned the genotype XIII. The genetic distances between 13 genotypes were 0.17-0.41, higher than those within genotypes, and the genetic distances between genotype XIII and other 12 genotypes were also 0.21-0.40. Unfortunately, due to few sequences of VP1/2A region available in GenBank database, other Chinese CVA9 strains were unable to analyze for genotyping.

In this study, the RGD motif within the C-terminal extension of the VP1 capsid protein of CAV9 [13] was also found to be fully conserved among 13 CAV9 genotypes including strain Gansu05-1/GS/CHN/2005 which suggests that the motif, although not essential for virus viability in cell culture conditions, plays a vital role in replication of the virus in humans.

## Discussion

Enteroviruses represent the main etiological agents of epidemics of viral meningitis and especially the serotypes related to the HEV-B species, and more than 50% of meningitis cases were due to echovirus 30 [14]. In 2005, outbreak of aseptic meningitis occurred in Tianshui city of Gansu Province, China. The results of the present study indicated that CVA9 was mainly responsible for this outbreak. This postulation is supported by the clinical manifestations of the affected patients, epidemiological data of the outbreak, the results of RT-PCR and sequence determination, conventional neutralization assays, IgM serological assays, viral isolation and phylogenetics analysis.

CVA9 is a member of the A subgroup which exhibits the typical pathogenicity in newborn mice [15], while the disease pattern in humans is more closely related to CVB infections. CVA9 is also one of the enteroviruses which may be associated with aseptic meningitis, and clinical CVA9 infections are common [16,17].

The genetic heterogeneity of enteroviruses makes it difficult to develop molecular typing that is both reliable and easy to perform. The genomic sequence encoding the VP1 capsid protein gives excellent results because of the high correlation between serotype and genetic information, and the availability of a complete database of reference strains isolated in the 1950's and 1960's. In this study, the pathogen was identified as CVA9 based on the complete VP1 gene. Through phylogenetic analysis and homogeneity analysis for partial VP1 gene, the strain Gansu05-1/GS/CHN/2005 shared highly homology with strains 97186/SD/CHN/1997/CA9 from Shandong province, belonging to different lineages with other 11 Chinese CVA9 strains. It indicated that there were multiple transmissions chains of CVA9 circulation in different provinces in China, and the similar virus were also found in Japan.

However, it is difficult for systematic genotyping based on VP1 gene because few complete CVA9 VP1 sequences available in the world. Santti *et al*. divided CVA9 strains into 12 genotypes using the criteria designated for PVs that strains sharing >85% identity in the VP1/2A junction region belong to the same genotype [18]. By compared this region of the CVA9 genome, it was found that strain Gansu05-1/GS/CHN/2005 isolated in this outbreak might be a new genotype of related sequences based on this criteria and could be assigned for genotype XIII. It seemed that this genotype virus circulated in China in recent years. So far, a number of genetically distinct genotype of viruses may currently circulate in the world. The identification of a new CVA9 genotype in China was useful for identifying the source, tracking transmission pathways and documenting changes in the viruses present in particular regions over time. However, due to no other Chinese CVA9 sequences of VP1/2A was available to analyze for genotyping, it is very difficult to know the time of this new genotype occurred, and whether co-circulated of other CVA9 genotypes existed.

In recent years, CVA9 was also isolated in many other countries including Spain, Finland, Canada, Netherland and United States etc through routine diagnosis for clinical samples from patients suspected viral central nervous system infection [10,19]. In all genotypes of CVA9, most of the strains within each genotype showed geographical clustering; evidence of long-distance importation of virus strains was also reported [10]. However, because of the limit data of Chinese CVA9 strains available, it was very difficult to clarify the molecular epidemiology of CVA9 in China.

In conclusion, this is the first reporting the outbreak of aseptic meningitis caused by CVA9 in China, and a new genotype of CVA9 was found based on the sequences of VP1/2A region. However, further analysis is needed to investigate the evolution of the CVA9 by obtained more sequences of virus strains and to find out their genetic relationships, and molecular epidemiological surveillance of CVA9 should also be strengthened.

## Conclusion

An outbreak of aseptic meningitis occurred in Tianshui city of Gansu Province, the People's Republic of China in 2005. In this study, CVA9 was confirmed as the pathogen responsible for this outbreak. The phylogenetic analysis indicated that the CVA9 strains isolated in this outbreak might belong to a new genotype.

## List of abbreviations used

CVA9: Coxsackievirus A9; RT-PCR: reverse transcription-polymerase chain reaction; CSF: cerebrospinal fluid; HEV: Human Enteroviruses; CPE: cytopathic effect; IgM: immunoglobulin M.

## Competing interests

The authors declare that they have no competing interests.

## Authors' contributions

ALC, ZZ, WBX prepared manuscript. WBX designed the study and organized the coordination. ALC performed data analysis. ALC, DSY, ZZ, LM, HL, JFL, GYL collected specimens and performed RT-PCR, virus isolation, viral identification, Coxsackievirus IgM assays and neutralization test. All authors read and approved the final manuscript.
